# A Case of Rectal Malakoplakia Associated With Crohn’s Disease: An Incidental Finding

**DOI:** 10.7759/cureus.84829

**Published:** 2025-05-26

**Authors:** Jean C Lafontaine Álvarez, Gabriela Portilla Skerret, María J Marcos Martínez, Rafael Rodríguez López

**Affiliations:** 1 Pathology and Laboratory Medicine, University of Puerto Rico, Medical Sciences Campus, San Juan, PRI; 2 Gastroenterology, University of Puerto Rico, Medical Sciences Campus, San Juan, PRI

**Keywords:** crohns, diseases of the colon and rectum, gastro-intestinal, gram negative, malakoplakia

## Abstract

Malakoplakia is a rare granulomatous disorder characterized by defective phagolysosomal activity in macrophages and the presence of Michaelis-Gutmann (MG) bodies, often affecting immunosuppressed individuals. Although it most frequently involves the genitourinary tract, gastrointestinal involvement - particularly in the rectum and sigmoid colon - has been documented. We report a rare case of rectal malakoplakia in a 28-year-old female with penetrating ileocolonic Crohn’s disease (CD) on infliximab. During surveillance colonoscopy, distal rectal mucosal nodularity, erythema, and friability were noted. Histopathology showed foamy histiocytes containing periodic acid Schiff-positive, diastase-resistant MG bodies, and CD68 confirmed histiocytic origin. A diagnosis of rectal malakoplakia was rendered, and the patient was managed with ciprofloxacin while continuing immunosuppressive therapy and a scheduled follow-up colonoscopy.

Malakoplakia may mimic malignancy or inflammatory bowel disease clinically and endoscopically, making histologic evaluation essential for accurate diagnosis. *Escherichia coli* is the most commonly implicated organism, and fluoroquinolones are typically effective treatments. Surgical intervention is generally reserved for refractory cases. This case highlights the need to consider malakoplakia in the differential diagnosis of atypical rectal lesions in immunosuppressed patients, including those with CD, to ensure timely and appropriate management.

## Introduction

Malakoplakia is an uncommon granulomatous disease of macrophage dysfunction, most frequently involving the genitourinary tract, but it has also been reported in the gastrointestinal (GI) system - particularly the rectum and sigmoid colon [1,2]. The term “malakoplakia” originates from the Greek words “malakos” (soft) and “plakos” (plaque), describing the gross appearance of the lesions as soft, plaque-like structures. Histologically, it is characterized by Michaelis-Gutmann (MG) bodies - concentrically layered, mineralized bacterial remnants within histiocytes - that serve as a pathognomonic feature [3]. Although malakoplakia can occur in immunocompetent individuals, it is more commonly observed in immunocompromised patients, including those with malignancy, acquired immune deficiency syndrome/human immunodeficiency virus (HIV/AIDS), autoimmune disorders, and chronic infections [4-6].

The pathogenesis of malakoplakia is not completely understood. Proposed mechanisms include impaired bacterial digestion due to defective phagolysosomal activity in macrophages, often associated with decreased levels of intracellular cyclic guanosine monophosphate (cGMP), and impaired lysosomal enzyme release [3,5]. This results in undigested bacterial debris accumulating and eventually calcifying with iron and calcium deposits [3].

Clinically, GI malakoplakia may present with nonspecific symptoms such as abdominal pain, diarrhea, weight loss, or rectal bleeding. These manifestations overlap significantly with other GI conditions, including malignancy and inflammatory bowel disease (IBD), often complicating the diagnosis [4,7-10]. Endoscopically, malakoplakia may resemble neoplastic or ulceroinflammatory lesions, further contributing to potential misdiagnosis [4,9].

Due to the rarity of malakoplakia in the GI tract, particularly in the rectum, its exact incidence is not well established. Only isolated case reports and small case series are available in the literature, making it difficult to assess consistent patterns or associations with conditions such as Crohn’s disease (CD) [1,4,11]. Nonetheless, some authors have proposed that chronic mucosal inflammation and immunosuppressive agents may contribute to the development of malakoplakia in this population [4,11].

Herein, we present a rare case of rectal malakoplakia, discovered incidentally during surveillance colonoscopy in a young female with fistulizing ileocolonic CD under infliximab therapy. This case highlights the diagnostic challenges and therapeutic considerations of malakoplakia in IBD.

## Case presentation

A 28-year-old female with a history of penetrating ileocolonic CD for the last three years presented to the gastroenterology clinic for routine follow-up and surveillance colonoscopy. Her clinical course was notable for multiple abdominal and pelvic surgeries, including perianal abscess drainage, colo-ovarian and small bowel fistula repair, appendectomy with Hartmann’s procedure, and creation of a diverting colostomy. She was undergoing maintenance immunosuppressive therapy with infliximab due to the aggressive phenotype of her CD.

On colonoscopic examination, the mucosa from the proximal rectum to the cecum appeared grossly normal. In contrast, the distal rectum demonstrated focal mucosal nodularity, superficial ulceration, erythema, and friability - features raising suspicion for active inflammation, dysplasia, or neoplasia. Targeted biopsies were obtained from this area.

**Figure 1:**
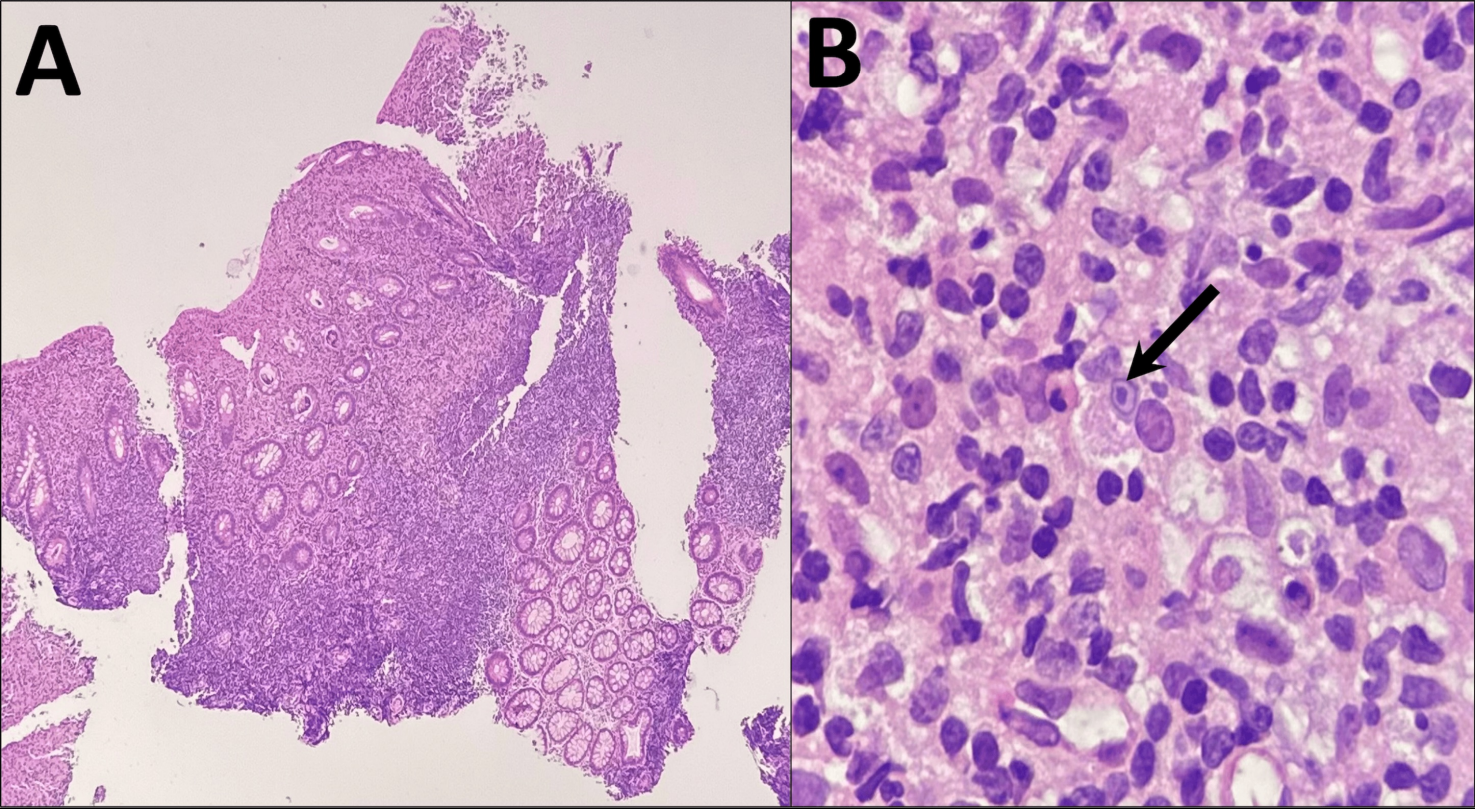
Histopathologic findings from distal rectum biopsy (H&E) A) 40x: There is crypt distortion and irregularity, with diffuse infiltration of the lamina propria, predominantly by numerous foamy epithelioid histiocytes, but also lymphocytes, plasma cells, and neutrophils. B) 600x: Occasional Michaelis-Gutmann bodies were seen within the cytoplasm of foamy epithelioid histiocytes (black arrow).

Histopathologic examination revealed crypt distortion with diffuse lamina propria infiltration by large, foamy epithelioid histiocytes (Hansemann cells), admixed with lymphocytes, plasma cells, and neutrophils (Figure 1A). On high-power magnification, occasional MG bodies were observed (Figure 1B). These basophilic, concentrically laminated, targetoid inclusions indicate mineralized bacterial remnants within phagolysosomes and are pathognomonic for malakoplakia [3,5].

**Figure 2:**
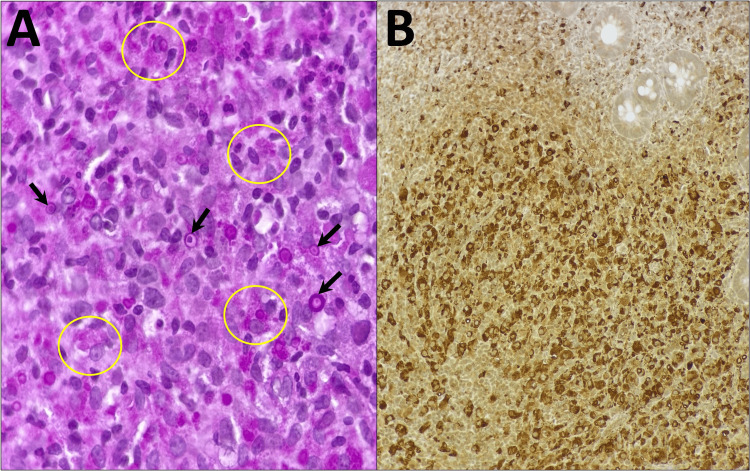
Ancillary studies A) 600x: Periodic acid-Schiff stain demonstrating accumulated Hansemann histiocytes with abundant 5-10 µm granular, basophilic cytoplasmic inclusions (yellow circles), and numerous Michaelis-Gutmann bodies (black arrows). B) 200x: Immunohistochemistry stain positive (+) against CD68, highlighting numerous foamy epithelioid histiocytes infiltrating the lamina propria.

Ancillary studies confirmed the diagnosis. Periodic acid-Schiff (PAS) staining highlighted numerous PAS-positive, diastase-resistant inclusions within the cytoplasm of histiocytes (Figure 2A). Immunohistochemistry for CD68 (monoclonal antibody, clone KP-1) showed strong cytoplasmic positivity in the foamy histiocytes, confirming their macrophage lineage (Figure 2B) [3,12]. No granulomas, epithelial dysplasia, or malignancy were identified in the sampled tissue.

Based on these histological and immunohistochemical findings, a diagnosis of rectal malakoplakia was rendered. The patient was started on oral ciprofloxacin, a fluoroquinolone antibiotic with excellent intracellular penetration and known efficacy against *Escherichia coli*, the most commonly implicated organism in malakoplakia [2,5]. Given the necessity for continued immunosuppression due to her aggressive CD phenotype, infliximab therapy was maintained. She was referred to the infectious disease service for further evaluation and was scheduled for a follow-up colonoscopy to assess treatment response and disease progression.

## Discussion

Malakoplakia is a rare granulomatous condition that most frequently affects the genitourinary tract; however, GI involvement, particularly in the rectum and sigmoid colon, has also been increasingly recognized [1,2]. Within the GI tract, rectal malakoplakia is uncommon and often discovered incidentally during evaluation for other suspected conditions [5,11]. The disease is typically associated with immunosuppressed states or chronic systemic illnesses, including malignancy, HIV/AIDS, diabetes mellitus, and IBD [4,6,7,13].

While malakoplakia and CD both involve granulomatous inflammation, their coexistence has not been previously described, and no established pathophysiologic connection is currently recognized. Chronic intestinal inflammation and immunosuppressive therapies, such as tumor necrosis factor (TNF) inhibitors, may impair macrophage function and increase susceptibility to bacterial persistence and defective intracellular clearance, potentially predisposing patients to malakoplakia [4,12]. In the present case, the coexistence of a fistulizing CD phenotype and long-term infliximab therapy may have created an immunologic environment conducive to developing this rare condition.

On histologic examination, the hallmark of malakoplakia is the presence of MG bodies - mineralized, concentric inclusions within sheets of foamy macrophages (Hansemann cells) - a pathognomonic feature that aids in distinguishing it from other granulomatous conditions [3,5]. These inclusions are typically PAS-positive and diastase-resistant, and CD68 immunohistochemistry confirms the histiocytic origin [3,12]. Identifying MG bodies is critical, as malakoplakia clinically and endoscopically mimics malignancy, tuberculosis, and active IBD [4,9,10].

In our patient, colonoscopy revealed mucosal nodularity, superficial ulceration, and friability in the distal rectum - nonspecific findings that warranted histologic evaluation. The biopsy findings were essential in distinguishing malakoplakia from more common CD-associated pathology and guiding appropriate management. This mirrors previously reported cases, in which malakoplakia was incidentally identified during investigations for unrelated or overlapping conditions such as malignancy [4,5,11,14].

Management of malakoplakia focuses on bacterial eradication and immunomodulation. Fluoroquinolones, such as ciprofloxacin, are the cornerstone of therapy due to their high intracellular concentrations and effectiveness against *E. coli*, the most commonly implicated pathogen [2,5]. In select cases, cholinergic agents like bethanechol have been used to increase intracellular cGMP levels and restore phagolysosomal function in macrophages [8]. Surgical resection is generally reserved for patients who fail medical therapy or develop complications [2,5]. In our case, the patient responded favorably to oral ciprofloxacin, and infliximab therapy was continued with close monitoring, given the aggressive phenotype of her CD.

This case highlights the importance of including malakoplakia in the differential diagnosis of colorectal lesions, particularly in immunocompromised patients or those receiving biologic therapy. Early histopathologic recognition can prevent misdiagnosis, avoid unnecessary surgical intervention, and enable effective, targeted antimicrobial therapy. Given the rarity of GI malakoplakia - especially in CD - additional research is needed to better define its pathogenesis, clinical course, and optimal management strategies.

## Conclusions

Rectal malakoplakia is a rare histopathologic diagnosis that can mimic malignancy or active IBD, particularly in immunosuppressed individuals or patients with chronic GI conditions like CD. Accurate diagnosis hinges on histologic evaluation, as clinical and endoscopic findings are often nonspecific. In this case, timely recognition enabled targeted antibiotic therapy and helped avoid unnecessary escalation of immunosuppression or surgical intervention.

The coexistence of malakoplakia and CD presents diagnostic and therapeutic challenges, particularly given the lack of standardized management guidelines. This case contributes to the limited body of literature on GI malakoplakia in the setting of IBD, reinforcing the importance of histopathologic evaluation in distinguishing it from other rectal lesions. In immunocompromised individuals, or those with longstanding IBD, prompt biopsy of atypical mucosal findings is essential to avoid misdiagnosis. This case underscores the need for greater clinical awareness and further research to clarify underlying pathophysiologic mechanisms, improve diagnostic accuracy, and guide more effective, evidence-based treatment strategies.

## References

[1] Liu X, Yu C, Zhao Z, Zheng Y, Chen X, Zhou D (2023). Rectal malakoplakia mimicking advanced rectal cancer: a case report. Heliyon.

[2] Prakash A, Noor N, Marchant A, Ghori M, Molani RA (2024). Colonic malakoplakia. J Ayub Med Coll Abbottabad.

[3] Hyun KH, Shin HD, Kim DH (2013). Malakoplakia in a healthy young female patient. Korean J Intern Med.

[4] Lee M, Ko HM, Rubino A, Lee H, Gill R, Lagana SM (2020). Malakoplakia of the gastrointestinal tract: clinicopathologic analysis of 23 cases. Diagn Pathol.

[5] Achufusi TG, Jessamy K, Chebaya P, Rawlins S (2020). Rectal malakoplakia. Proc (Bayl Univ Med Cent).

[6] Yen JM, Soh NW, Petersson F, Pandya G (2017). Rectosigmoid malakoplakia. BMJ Case Rep.

[7] Trepeta SS, Trikha S, Alterman DD (1998). CT of colonic malakoplakia in a patient with AIDS. AJR Am J Roentgenol.

[8] Taher M, Shahsia R, Ebrahimi Daryani N (2021). Malakoplakia as a rare cause of diarrhea: case presentation and review of literature. Middle East J Dig Dis.

[9] Yared RA, Badran HA, Kamareddine MH (2018). Colonic malakoplakia: a rare finding in a healthy male. Case Rep Gastroenterol.

[10] Ma YC, Wang SW, Chang KC (2022). Colonic malakoplakia with retroperitoneal extension mimicking advanced colon cancer. J Gastrointest Surg.

[11] Yousef GM, Naghibi B, Hamodat MM (2007). Malakoplakia outside the urinary tract. Arch Pathol Lab Med.

[12] Zanelli M, Ragazzi M, Serra S, Bellafiore S, Ascani S, De Marco L (2016). Malakoplakia associated with multiple adenomas of the colon: an extremely rare incidental finding. Int J Surg Pathol.

[13] Wang Z, Ren J (2022). Clinical analysis of renal failure caused by malakoplakia: a case report and literature review. Front Med (Lausanne).

[14] Cipolletta L, Bianco MA, Fumo F (1995). Malacoplakia of the colon. Gastrointest Endosc.

